# 6-minute walk test as a measure of disease progression and fatigability in a cohort of individuals with RYR1-related myopathies

**DOI:** 10.1186/s13023-018-0848-9

**Published:** 2018-07-03

**Authors:** Jessica W. Witherspoon, Ruhi P. Vasavada, Melissa R. Waite, Monique Shelton, Irene C. Chrismer, Paul G. Wakim, Minal S. Jain, Carsten G. Bönnemann, Katherine G. Meilleur

**Affiliations:** 1 National Institute of Nursing Research, NIH, Bethesda, MD USA; 2 Rehabilitation Medicine, NIH, Bethesda, MD USA; 3 National Institute of Neurological Disorders and Stroke, NIH, Bethesda, MD USA; 4 Biostatistics and Clinical Epidemiology, NIH, Bethesda, MD USA

**Keywords:** RyR1, 6-min walk test, Disease progression, Fatigue

## Abstract

**Background:**

*RYR1*-related Myopathies (*RYR1*-RM) comprise a group of rare neuromuscular diseases (NMDs) occurring in approximately 1/90000 people in the US pediatric population. *RYR1*-RM result from pathogenic mutations in the ryanodine receptor isoform-1 (*RYR1*) gene where consequent RyR1 protein calcium dysregulation leads to impaired excitation-contraction coupling, oxidative and nitrosative stress, and mitochondrial depletion. These physiological deficits perpetuate RyR1 dysfunction causing further muscle injury, muscle weakness, and muscle fatigue. Muscle weakness and fatigue are two primary complaints in patients with *RYR1*-RM and are major symptoms that limit the ability of individuals to perform activities of daily living. The six-minute walk test (6MWT) is an endurance test with high reliability and validity used to measure walking capacity, disease progression, and more recently, fatigability in NMDs with limited results in *RYR1*-RM. Therefore, the purpose of our study is to objectively assess disease progression and fatigability in *RYR1*-RM affected individuals using the 6MWT. We *hypothesized* that 6MWT distance and fatigability would not change significantly between 0 and 6-month visits in *RYR1*-RM patients, given the clinically reported stable or slowly progressive nature of the disease. We also *hypothesized* participants would show fatigability during the 6MWT, given muscle weakness and fatigue are the two primary complaints of affected individuals.

**Results:**

As expected, paired t-test analyses revealed no significant difference between total distance traveled (*p* = .608) or percent change in speed (*p* = .141) at 0-months compared with the 6-month visit. Fatigability was observed given the decline in speed between the first and last minute of the 6MWT at the 6-month time point (*p* ≤ .0005,). Although this decline was not significant at baseline, a significant decline in speed from the 1st minute did occur at minutes 2, 3, and 4 during the baseline visit.

**Conclusion:**

In this RYR1-RM cohort, the 6MWT showed disease stability over a 6-month period but revealed fatigability during the test. Given these results, the 6MWT may be a promising endpoint for evaluating fatigability and therapeutic efficacy in the 6-month treatment phase of our current n-acetylcysteine trial in this population. Improvement post intervention could be attributed to the intervention rather than variability in disease progression.

**Trial Registration:**

Clinical Trials.gov, NCT02362425, Registered 13 February 2015-Prospectively registered.

## Background

Affecting 1/90,000 children in the United States (US) [1], *RYR1*-related myopathies (*RYR1*-RM), though rare, comprise the most common congenital myopathies [2] in the US. *RYR1* is one of the largest genes in the human genome and encodes the major calcium channel in skeletal muscle, ryanodine receptor isoform-1 (RyR1). Numerous variants in *RYR1* have been identified as the cause of *RYR1*-RM subtypes, including central core disease (CCD), multi-mini core disease (MmD), centronuclear myopathy (CNM), core-rod myopathy, and congenital fiber type disproportion (CFTD).

*RYR1* mutations yield RyR1 protein dysfunction and thus calcium dysregulation, resulting in impaired excitation-contraction coupling and excessive mitochondrial oxidative stress [3, 4]. Affected individuals present with combinations of delayed motor milestones, hypotonia, fatigue, extremity muscle weakness, joint contractures, progressive scoliosis, susceptibility to malignant hyperthermia (MH), and, in more severe cases, ophthalmoplegia and/or respiratory failure [3, 5, 6]. *RYR1*-RM-associated symptoms may impair quality of life, especially in severely affected individuals, who are at risk for early mortality. Muscle weakness and fatigue are the two primary complaints in patients with *RYR1*-RM and are major symptoms limiting the ability of individuals to perform instrumental activities of daily living [7–9]**.** De Vries et al. noted [8], “fatigue accounts for an important part of the burden experienced by patients with neuromuscular disorders [8].”

The 6-min walk test (6MWT) is an endurance test with high reliability and validity [10] that allows for the assessment of walking capacity, disease progression, and treatment efficacy [11]. Recently [10, 11], the 6MWT was shown to serve as a measure of fatigability in neuromuscular diseases (NMDs) as determined by a decline in speed [10, 11]. Andersen et al. observed a 4.6% decline in speed (meters/second) between first minute and last minute (minute 6) of the 6MWT in patients with various NMDs [11]. Currently, the 6MWT is not only the preferred measure of walking capability and endurance, but also of fatigability in adult and pediatric neurological and neuromuscular disorders [12]. It has been used as the primary outcome measure in clinical trials, which led to its approval as one of the preferred walking test measurements [13, 14]. However, the 6MWT has not yet been objectively studied as an outcome measure in *RYR1*-RM affected individuals.

The clinical impression of *RYR1*-RM suggests a non-progressive (static) [15, 16] or slowly progressive [16] group of diseases. However, disease progression has likewise not been formally studied. It remains undetermined whether *RYR1*-RM would remain stable or progress over the time frame of a typical clinical trial. The purpose of our study was to objectively assess disease progression and fatigability in individuals with *RYR1*-RM using the 6MWT over 6 months, in preparation for future clinical trials.

## Results

### 6MWT

#### Disease progression

**Fig. 1:**
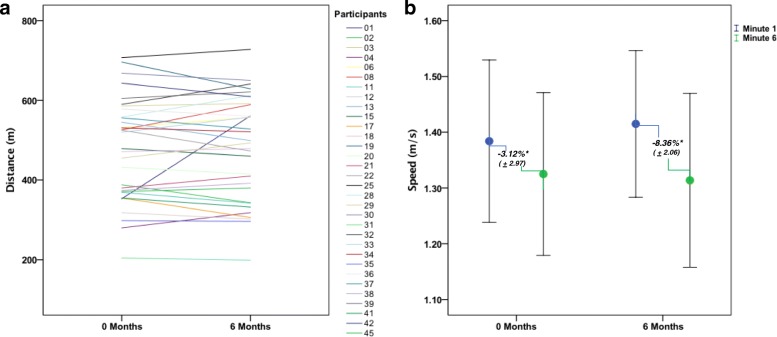
Disease Progression. **a** Total 6-min walk distance at 0 and 6-month visits by participant. This figure includes only the 32 participants with both 0- and 6-month data. **b** Mean walk speed (95% CI) at minutes 1 and 6 at 0 and 6 month visits. Percentage values represent the % change between minute 1 and minute 6 for each visit. *significance

RYR1-RM participants, on average, performed ~ 79% (range 32.2–119%) of the predicted norm for their age, sex, and height on the 6MWT [17–19]. This average did not change between the baseline (79.1% ± 3.55) and 6-month (79.9% ± 3.72) visits. The mean distances walked at baseline (477 m ± 22.8) and 6 months (481 m ± 23.2) were also compared. Paired t-test analysis showed there was no evidence of change in percent of the predicted norm (*p* = .612) or distance walked between visits (*p* = .608). Similarly, the percent change in walking speed between the first and 6th minute did not change between visits; *p* = .141. Figure 1a compares total distance walked at baseline and 6-month visits per individual. For each visit, the mean walking speed and the mean percent change in walking speed between minutes 1 and 6 are shown in Fig. 1b.

#### Fatigability

**Fig. 2:**
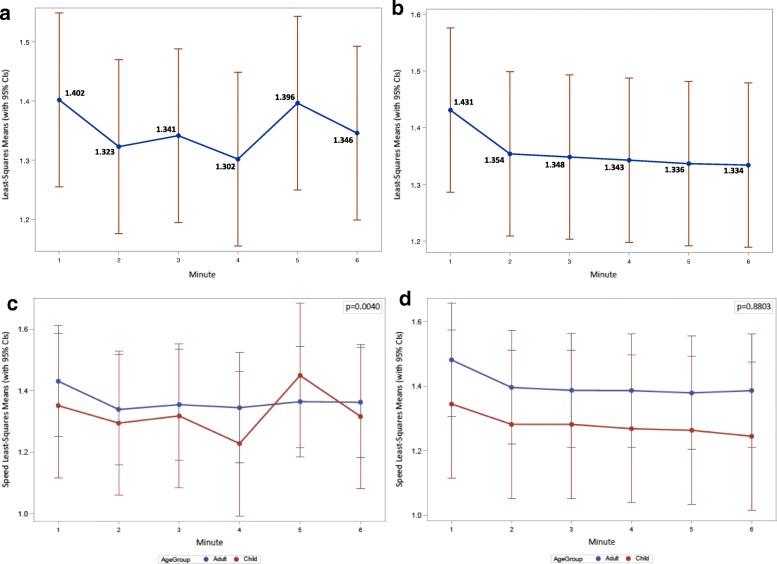
Least square means for walking speed at each minute during 6MWT for baseline (**a**) and 6 month (**b**) visits. Comparison of least square means for walking speed at each minute during 6MWT between adults and children at baseline (**c**) and 6-month (**d**) visits

Paired t-test analyses revealed no significant difference between percent change in speed between the 0-month and 6-month visits. However, a decline in speed between the first and last minute of the 6MWT was observed. As depicted in Fig. 2a, mean walking speeds during the 6MWT were variable at the baseline visit: the average speed started to plateau after the 2-min interval, increased at the 5-min interval, and then returned to the 2-min interval speed. In contrast, mean walking speeds declined and gradually plateaued throughout the test when performed at 6 months.

At the baseline visit, the fastest walking speed was in the first minute. Minutes 2–6 showed variations in speed, with the slowest minute being minute 4. Although walking speed was variable during the baseline visit, the speeds at minutes 2 (*p* = .003), 3 (*p* = .006), and 4 (*p =* .0001) were each significantly slower than the speed walked in the first minute. These *p*-values are unadjusted for multiplicity.

Similar to the baseline visit, the 1st minute of the 6-month visit 6MWT was the fastest. After the first minute a plateau in speed was observed (Fig. 2b). There was a significant (*p* ≤ .0005) decrease in walking speed between the first minute and each minute interval thereafter (mins 2–6). As expected, the observed change (decrease) in distance traveled between the first and last minute of the 6MWT was reflected in the change (decline) in speed between the first and last minute of the 6MWT.

The speeds of adults and children were also compared. At both month 0 and month 6, the average speeds across the 6 min were not statistically different between the two age groups (month 0, *p* = .782; month 6, *p* = .389). Only at minute 5 of month 0, did the average speed of children exceed that of adults, hence the interaction between minute intervals and age group (Fig. 2c). In all other minutes and in both month 0 and 6, the speed for adults was slightly higher than that of children, but again the difference was not statistically significant (Fig. 2c and d).

**Fig. 3:**
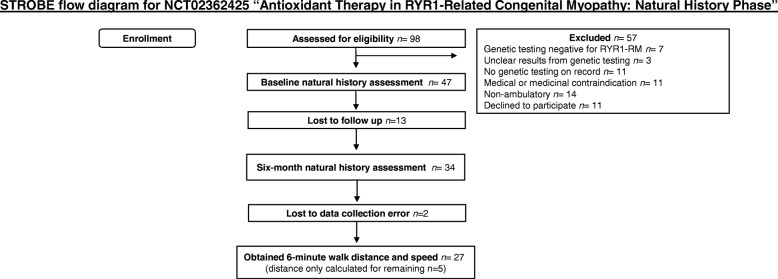
STROBE Flow diagram for participants included in the natural history phase of the RYR1 NAC trial

Interestingly, the speed at the 2-min interval was comparable to the speed at the 6-min interval at both study visits. The initial decline in speed from the first to the second minute was nearly comparable to the decline in speed from the 1st-minute interval to the 6th-minute interval. In contrast, no significant difference was observed at either visit between the speed at 2 min and at 6 min (baseline visit: *p* = .875, 6-month: *p* = .161). Given that researchers are currently assessing the possibility of implementing a 2-min walk test (2MWT) instead of the 6MWT, fatigability in addition to endurance may be another important variable to assess during a 2MWT in the NMD population.

## Discussion

The 6MWT originated as a way to measure functional capacity and endurance in people with cardiorespiratory disease, for which normative values have been established [20]. Now, the 6MWT is widely used across diseases, including NMDs [11, 20–22]. Andersen et al. reported using 6MWD in NMDs, including myotonic dystrophy type 1, facioscapulohumeral dystrophy, sporadic inclusion body myositis, Kennedy disease, Limb-girdle muscular dystrophy, Charcot Marie-Tooth, mitochondrial myopathy, and mixed myopathies. The average distance ambulated across all of the above-mentioned diseases was 405 m (range 65-750 m).

More recently, the 6MWT has also been employed as a measure of fatigability in multiple sclerosis (deceleration index depending on disease severity) [23], myasthenia gravis (degree of deviation from stable performance using linear trends) [24], and the aforementioned NMDs (percent decrease between 1st and 6th minute) [11]. Fatigability is defined as an objective measurement of decline in performance [24].

This natural history study assessed disease progression and fatigability in an RYR1-RM cohort over a 6-month time course in preparation for future clinical trials. Our study showed there was no significant change in the mean distance walked or mean walking speed during the 6MWT over a 6-month time frame, confirming the stable or slowly progressive nature of *RYR1*-RM, despite having a phenotypically heterogeneous cohort. Although this study was of shorter duration than most natural history studies, it provides a glimpse into the progression of *RYR1*-RM during a 6-month time frame. If an intervention were to show improvement in *RYR1*-RM within this time frame, this change could be attributed to the intervention rather than disease progression. A clinically meaningful difference of 28.5 m has been established in boys with Duchenne muscular dystrophy older than 5 years of age [25], but no such difference has been established in individuals affected with *RYR1*-RM.

Given that our results demonstrated *RYR1*-RM disease stability using the 6MWT over a course of 6 months and this test is accepted as a clinically meaningful outcome measure in various NMDs [21], the 6MWT may serve as a promising endpoint for evaluating therapeutic efficacy in the treatment phase of current and future trials. Our results also indicate that walking speed may potentially be a clinically meaningful outcome in *RYR1*-RM. The decrease in walking speed observed in these RYR1-affected individuals between minutes 1 and 6 could be impacted by a treatment. In other diseases, an improvement of walking speed of 0.1–0.2 m/s has been reported as the minimal clinically important difference (MCID) [26]. Using this information and our 6MWT data in *RYR1*-RM affected individuals, only 10 people would be needed to power a trial at 90% with an alpha of .05 if change in walking speed were the primary outcome based on an MCID of 0.2 m/s.

Compared with the general NMD population [11], participants with *RYR1*-RM had similar walking speeds to individuals with Becker Muscular Dystrophy (1st min: 1.43 m/s, 6th min: 1.36 m/s). *RYR1*-RM affected individuals began the test with the fastest 6-min walk speed (1.43 m/s), but also exhibited the greatest decline in speed (1.33 m/s) at the end of the test, which was observable starting at the 2-min interval. In our cohort, individuals with *RYR1*-RM demonstrated variable speeds at minute intervals at the baseline visit. In contrast, at the 6-month visit, there was an initial decline in speed that gradually plateaued after the 2nd minute. The fastest speed was achieved in the first minute at both baseline and 6-month visits. Because the variability in speed was observed at baseline but not at the 6-month visit, this variability could be attributed to a learning effect, which is common in other NMDs [27, 28]. Utilizing standardized 6-min walk testing methods, our findings demonstrated a decline in performance at both time points, which suggests that individuals with *RYR1*-RM exhibit fatigue during the 6MWT. A decline in speed between the first and 6th minute was also accompanied, as would be expected, by a decrease in distance traveled, reflecting a decline in performance by the end of the test.

To the best of our knowledge, a single study has assessed the percent decline in NMDs compared with the healthy population to date [11]. However, standard error values were not provided, and thus we were unable to directly compare the average percent decline between their study and ours. The reported percent decline in walking speed for the group of NMDs (myotonic dystrophy, limb girdle muscular dystrophy, fascioscapulohumeral dystrophy, Kennedy disease, Charcot Marie-Tooth neuropathy, mitochondrial myopathy, sporadic inclusion body myositis, Becker muscular dystrophy, and a mixed group of myopathies including congenital myopathy) was 4.6% between the first and last minute [11]; individuals in our *RYR1*-RM cohort presented with a mean percent decline in speed of 3.12% (± 2.97) at baseline and 8.36% (± 2.06) at the 6-month visit. The previously reported healthy population demonstrated a 1.4% decline in walking speed over the 6 min [11]. Percent decrease in speed may be clinically meaningful in identifying fatigue-related functional capacity in *RYR1*-RM, but this would require a larger 6MWT study including both *RYR1*-RM and healthy participants.

There has been a growing interest in comparing the two-minute walk test with the 6MWT [29]. Interestingly, a decline in speed in our cohort initially occurred at the 2-min interval of the 6MWT for the baseline and 6-month visits. This decline in speed from the first minute interval to the 2nd minute interval was not significantly different from the decline in speed between the first and 6th minute intervals. Given our study shows that fatigability can be captured within the first 2 min of the 6MWT, this may further support the use of a 2MWT but needs to be explored further.

Since the 6MWT detects a decline in walking speed in *RYR1*-RM, prospectively studying cardiorespiratory measures of fatigue during the 6MWT could provide insight into the cardiorespiratory response of individuals with *RYR1*-RM. In people with cystic fibrosis and chronic obstructive pulmonary disease, a decline in speed during the 6MWT with an increase in HR, oxygen saturation, and dyspnea has been observed [30]. However, cardiorespiratory fatigue response may not be achieved in people with *RYR1*-RM given that they do not have primary lung disease and local muscle fatigue may occur first in myopathy patients.

### Study limitations

Given the rare nature of this disease, a few limitations to our study include the small sample size and varied disease expression among participants, which limits our ability to categorize by severity. Thus, we were unable to observe whether performances varied by disease severity or by the presence of neuromuscular issues that limited walking in some cases. We are currently exploring criteria for categorizing disease severity. Also, the differences in intermittent vs gradual decline in speed at the baseline versus 6-month visits could be due to a learning effect or to changes in individuals’ efforts. This may be addressed in the future by having a practice 6MWT with a period of rest.

## Conclusion

The 6-min walk test has been used to assess endurance, ambulatory capacity, and cardiopulmonary function for a range of diseases for many years. More recently, it has been used to assess fatigability in neuromuscular disorders. This study was the first to evaluate 6-min walk distances and speeds in individuals with *RYR1*-RM in order to assess disease progression and fatigability over a course of 6 months. Our findings confirm clinical reports of *RYR1*-RM as being a group of stable or slowly progressive diseases. The significant decrease in walking speed between interval minutes 1 and 6 observed at both baseline and 6-month visits indicates the 6MWT is an appropriate measure of fatigability in individuals with *RYR1*-RM. Based on our results and the ability to now properly power future studies using these data, assessing changes in speed during 6MWT intervals may serve as a valid outcome measure for use in future natural history studies and/or clinical trials, especially those addressing fatigability in this population.

## Methods

### Participants

Participants were enrolled in our *RYR1*-RM double-blind, placebo-controlled N-acetylcysteine (NAC) Trial (NCT02362425) at the National Institutes of Health (NIH). This clinical trial, consisting of both a natural history and a treatment phase, was approved by the NIH Combined Neurosciences Institutional Review Board. Participants (and/or caregivers) who agreed to participate signed informed consent forms (and assent if a minor under the age of 18 years). Recruitment for this trial began in January 2015. The natural history phase (0–6 months) of the clinical trial, reported here, was completed by 32 participants (13 males, 19 females) between March 2015 and November 2016 at the NIH Clinical Center in Bethesda, MD. Figure 3 shows the Strengthening the Reporting of Observational Studies in Epidemiology (STROBE) flow diagram for participants included in this paper. Of these participants, 12 were children with a mean age of 10.1 (± 2.70), and 20 were adults with a mean age of 39.4 (± 12.2). The cohort was comprised of CCD, MmD, and CNM, many with malignant hyperthermia susceptibility. Inclusion criteria for this study included ≥ 7 years of age, ambulatory without assistive devices or orthotics, and confirmed genetic diagnosis of *RYR1*-RM. Muscle biopsy was required initially, but, after six months, this criterion was changed to “preferred” to facilitate recruitment. Participants were excluded if they presented with a history of liver or lung disease, ulcers, dysphagia, were pregnant or breastfeeding, planned to become pregnant, or were consuming medicine that interacts with NAC or other antioxidants.

### 6MWT

The 6MWT was administered in a single day at the NIH Rehabilitation Medicine Department using the American Thoracic Society (ATS) guidelines [14]. It was conducted at baseline in the early afternoon on the second day of the visit and was repeated at 6 months on the second day of the visit at the same time as baseline. After arrival at the testing site, participants rested for five minutes. Blood pressure and resting heart rate were measured before exercise, immediately post-exercise, and after a 5-min recovery. All participants were instructed to walk back and forth along a 50 m hallway for 6 min, “as quickly and safely as possible.” During the test, to ensure safety of the participant, a clinician trailed slightly behind the subject to avoid introducing a pacing bias. The participants were given a series of small objects (e.g., beanbag toys) to be used as markers. Participants were asked to drop the marker at every minute interval to record his/her location at the end of each minute. If dropping the markers appeared to be distracting to a participant, an individual who served as a chaser dropped the markers instead. Participants were not permitted to sit or lean against the wall or a chair if they required a rest period during the test. If rest was required, the clinician continued to keep time. Verbal encouragement was given in accordance with ATS guidelines [14]. In addition, children who appeared to be distracted or slowing were given more frequent verbal encouragement. The distance walked at each minute interval was documented. The setting, procedure, and clinician remained consistent when possible at the follow-up visit (6-months) to decrease potential sources of bias. In three cases, an alternate clinician administered the 6MWT.

### Statistics

Data for the 6MWT were collected and stored in the NIH Clinical Trials Database, CTDB. Statistical analyses were performed using SAS v9.4 software. Descriptive analysis was used for sample demographics. Results are expressed as the mean ± SEM (Standard Error of the Mean). Differences in 6MWT distance performed at 0 months (baseline) and 6 months as well as the percent change in walking speed at baseline and 6 months were determined using paired t-tests to assess disease progression. A repeated-measures mixed model was fit to the data to assess fatigability based on changes in walking speed per minute during the 6MWT. An age effect was also introduced into the mixed model to determine if there was a difference in performance between adults and children.

A total of 32 subjects completed the 6MWT at 0 and 6-month visits. Of the 32 participants, 3 did not have a distance recorded for the first minute during their baseline visit, so changes in walk speed and distance could not be determined. One participant did not have properly recorded four and five-minute interval distances during their second visit. However, the total 6-min walk distance (6MWD) was recorded. For this reason, all 32 participants data were included in statistics for disease progression as determined by total walked distance during 6MWT, but data from only 27 participants were used for walking speed and fatigability-related statistics.

## Abbreviations

6MWD: 6-min walk distance
6MWT: 6-min walk test
ATS: American Thoracic Society
CCD: Central core disease
CFTD: Congenital fiber type disproportion
CNM: Centronuclear myopathy
MH: malignant hyperthermia
MmD: Multi-mini core disease
NAC: N-acetylcysteine
NMDs: Neuromuscular diseases
RyR1: Ryanodine receptor isoform-1
*RYR1*-RM: *RYR1*-Related Myopathies
